# Southern Branch, National Dental Association, Second Annual Meeting, New Orleans, La., February 9, 10, 11 and 13, 1899

**Published:** 1899-04

**Authors:** 


### PROCEEDINGS.

### Southern Branch, National Dental Association, Second Annual Meeting, New Orleans, La., February 9, 10, 11 and 13, 1899.

The second annual meeting of the Southern Branch of the National Dental Association was held in New Orleans, La., February 9, 10, 11 and 13, 1899.

The meeting was held in joint sessions with the Louisiana State Dental Society.

The venerable Presbyterian divine, Dr. B. M. Palmer, whose reputation for eloquence is National, opened the meeting with an invocation for God's blessing and guidance.

The Mayor of the city, Hon. W. C. Flower, was then introduced and tendered a warm and cordial welcome to the members of the Association.

Dr. Sarrazin welcomed the Southern Branch of the National in behalf of the Louisiana Society, in which he said that "we have succeeded in bringing here at the time of our annual meeting in New Orleans, the Southern Branch of the National Dental Association, is one of those events to which we can well look with satisfaction and pride, and too much credit for this achievement can not be given to our administration of the preceding year, which forsaw this possibility and took the initial steps in the movement. Again, we may justly congratulate ourselves that with a mere handful of workers in rank, we have succeeded in making such arrangements for the coming of this great and important convention as would throw no discredit upon our State in the eyes of these distinguished visitors from all over the country."

The response to these addresses was made by Dr. R. K. Luckie, of Holly Springs, Miss., on behalf of the Association. He said that from the tiresome work of daily routine life, the members had gladly come to attend this convention to be refreshed, benefited and encouraged by the deliberations of this convention. It was a pleasant duty that had been bestowed upon him to respond to the gracious, kind and cordial welcome extended by the distinguished Mayor of this city and the accomplished representative of the Louisiana State Dental Society—an organization which Dr. Sarrazin both honors and adorns. He felt that two such distinguished gentlemen truly represented the sentiments of the people for whom they had spoken. He paid a glowing tribute to New Orleans and to the State of Louisiana, which is known to have the finest quarantine station in the world, this port known to be the largest cotton market, this city the home of health, wealth, music and beauty, and the commercial gateway to the tropic seas, whose trade is to be enhanced by the opening of the canal, which will bring her into closer contact with the isles of the Pacific and the countries of the Orient. He referred with great fervor and eloquence to the fact that the American people are now held together by a closer bond of patriotic union than they ever were before.

Dr. Wm. Ernest Walker, of Pass Christian, Miss., the President of the Association, after a few preliminary remarks, proceeded to deliver the customary annual address.

Among other topics of general interest, Dr. Walker spoke as follows on the subject of the appointment of

DENTISTS IN THE ARMY AND NAVY.

He said: At our last annual meeting a committee was appointed to work in the interest of legislation in the matter of the appointment of dental surgeons to the army and navy. The subject was also brought before the National body at its meeting in Omaha, and a committee appointed to take charge of and have full control of the subject of legislation in this direction. A resolution was also adopted deprecating any independent effort in Congress on the part of other societies or individuals. I need not dwell upon the importance of the subject. Our soldier heroes on the battle field, in the camp, in the hospital, the men on board our war vessels bear witness that the hardships to which they were subjected were too often aggravated by the sufferings incident upon neglect of oral hygiene. Dr. Nicholas Senn, Lieutenant-Colonel in the United States Volunteers and chief of the operating staff with the army in the field, in his report on the surgery of Camp Wyckoff, says: "Of the organs frequently affected among the returning soldiers were the teeth. Patients suffering from carious aching teeth were numerous. In most instances they presented evidences of serious malnutrition, following disease and exposure; suppurated alveolitis was less frequent. Infection of many oral cavities showed that teeth had been sadly neglected during the campaign. I did all I could in the way of conserted dentistry by cleaning out cavities and packing with cotton, saturated with carbolic acid, but in the majority of cases the patients returned and insisted on having the painful teeth extracted." Much has been said in favor of attaching a dentist to each regiment to look after the teeth of men, and the observations made in Camp Wyckoff tend to support the propriety of such a much-needed addition to the medical service.

THE EDUCATION OF THE PUBLIC.

A topic worthy of your earnest consideration is that of the education of the public to an appreciation of the teeth as essential portions of the general economy, of the importance of preserving them in all their integrity and the means thereto. Individual instruction from the chair, while doing much good, still reaches only those who already have attained a degree of knowledge or appreciation along these lines. What is needed is to reach people before they need to visit our offices, and thus teach them by what means they may avoid the necessity of operative procedure. The most feasible plan thus far suggested appears to be through children in our public schools. In Mississippi, Alabama and Texas an effort is now being made to secure the co-operation of the State medical societies and the State school superintendents in enforcing an addition to the curriculum of either a special primer on oral hygiene, or an addition to the text-books, the use of which is already compulsory. I submit this idea for your consideration, with a hope that the plan may be encouraged by receiving your indorsement.

### LIFE INSURANCE EXAMINATIONS.

A no-less-important matter is that of the examination of the mouth by the medical examiners of life insurance companies, a subject which has been discussed by this body at our last meeting. Our corresponding secretary was instructed to initiate a correspondence with the officials of the leading life insurance companies of this country in an effort to ascertain their views on the subject. We look forward with interest to his report upon the subject, as its importance is of vital interest. The condition of the teeth in its bearing upon the general health constitutes such an important factor in the calculation of longevity that it is strange that it should have been so entirely overlooked in the matter of life insurance examination.

### THE JOURNAL.

I beg to enlist your interest in co-operation in the plan under consideration by the Association of which we are a branch, looking to the establishment of a journal which shall be the official organ of the dental profession—a journal in which shall be published the transactions of the National Association and its branches, and other original ideas pertaining to dentistry and the collateral sciences. At the meeting of organization at Old Point Comfort in 1897, a resolution was adopted to this effect,

Drs. John S. Marshall, J. Taft and L. G. Noel being appointed a committee to investigate the subject. The report of this committee, presented at Omaha by the chairman, Dr. Marshall, gave evidence of thorough investigation on various points, especially as to ways and means. The committee was continued for another year that it might work out the details of a scheme by which the committee thinks this very important work may be established and carried out. Their forthcoming report is looked forward to with interest, and there is no doubt but that in such competent hands the object will be accomplished, but the project is one that demands and deserves the support and co-operation of every member of this body, and I therefore beg to direct your attention to it.

### "ORTHODONTIA AND ORAL SURGERY."

BY G. E. HARDY, M.D., D.D.S., BALTIMORE, MARYLAND.

Dr. Hardy referred to Dr. Farrar's second volume as a masterpiece, the two volumes issued constituting the most comprehensive and complete treatise on orthodontia ever written. He spoke of the necessary close study of the models and of the patient, and of what has to be contended with in the mechanical and anatomical surroundings, and said, "eternal vigilance" is the price of success in regulating. Watch the appliance, watch the anchorage, watch the tooth to be moved and the teeth not to be moved, and watch the patient to see that the whole appliance is not removed from the mouth as soon as the patient reaches home.

Having tried various methods and "systems" for retracting the six anterior teeth, he had reached the conclusion that Angle's head-gear is the only thing that promises success.

He spoke of the "artistic treatment of natural teeth," as practiced by Dr. Younger, Dr. Payne and others, as a move in the right direction. The results obtained by the artistic sculpturing, carving and remodeling of badly-formed teeth are truly wonderful.

DISCUSSION.

Dr. Younger, being called upon, described what he considers the abnormal condition of the enamel surface, due to the contact of hard food substances, acids, etc. He practices removing this disintegrated surface layer, restoring and polishing the enamel to its original brilliancy and preventing decay which the roughened surface invites. In regard to retaining teeth which have been moved, he cited a case in which the tooth had a strong tendency to return to its original position—"a right superior central playing leap frog with the left"— whenever the retainer was removed. He finally, with a sharp bistoury, cut loose all attachment between the tooth and the tissues. By cutting loose the contracting tissue the tooth was no longer drawn out of place, and after wearing the retainer another week there was no further trouble.

Dr. Henry W. Morgan spoke of the importance of freeing teeth that have been moved from occlusion with the opposite teeth, unless this is such as to tend to hold them in the new position.

Dr. L. M. Cowardin spoke of a vicious habit that sometimes obtains, of so holding the tongue between or against the teeth as to interfere seriously with attempted regulation.

Dr. Walker spoke of a case in which, in 1888, between his own college terms, he had, in a case of marked hereditary prognathic development, after removing a bicuspid from each of the four quarters of the mouth, carried back simultaneously the six anterior teeth, superior and inferior *en bloc* in six months, using the patient's heavy suit of hair as a pad at the back of the neck to support a board which served as anchorage for the appliance placed upon the anterior teeth. He had succeeded in overcoming a hereditary deformity, enabling the patient (to whom ordinary conversation had been difficult from the fact that the lips could scarcely be made to cover the teeth) to become a successful school teacher with marked ability as an elocutionist.

### "CONSERVATIVE ANTRUM TREATMENT."

Dr. L. M. Cowardin read a paper opposing tooth extraction for the purpose of obtaining access to the central cavity and affording drainage, and cited cases successfully treated through the canine fossa without the sacrifice of a tooth.

DISCUSSION.

In the discussion of the paper, Dr. J. P. Corley described a case in which he had filled the buccal roots of a second molar and cemented a platinum cylinder in the palatine root, filling the cavity in the tooth around the cylinder with amalgam. Through this cylinder he was able to treat and drain the antrum and the case was discharged, cured, in two weeks. He objects to treating through the canine fossa, because of the obstruction offered by the soft tissues. He prefers to devitalize a tooth and treat through the root-canal.

Dr L. A. Smith described a case which he had successfully treated through a root-canal, subsequently placing a porcelain crown upon the root.

Dr. T. C. West described a case in which he inserted a silver drainage tube between a cuspid and bicuspid, making the end of the tube bonnet-shaped. He washed out the cavity with salt water and treated with iodoform vapor, using Blair's vaporizer.

Dr. B. Holly Smith opposed the method advocated in the paper and by the preceding speakers. He considered such operations too conservative. The opening should be such that the cavity can be freely explored with finger and thoroughly curetted. He considers entering and draining the antrum through the root of a tooth inexpedient and unwise, as being a method requiring longer time and more concentrated medicines than by other methods.

Dr. L. G. Noel spoke of the frequent complication with nasal catarrh, when the entire mucous membrane is involved in the trouble, and thought when it was a question between saving a tooth and being embarrassed by the presence of the tooth it was better to sacrifice even a sound tooth if that would give the assurance of satisfactory results. In treatment Dr. Noel uses Lugol's solution of iodin which is readily miscible with water and gives very beneficial results. He spoke of a condition sometimes existing in which the roots of a tooth may penetrate the floor of the antrum and be covered at the apex only by the periosteal membrane. In case of alveolar abscess the pus may lift the membrane until the entire antrum is invaded by accumulated pus, yet with no opening through the membrane. Dr. Noel does not believe in drainage tubes, but protects the opening into the antrum with a plate to prevent the ingress of food.

In closing the discussion Dr. Cowardin said that the paper did not advocate opening roots indiscriminately for the purpose of treating the antrum, neither was the method offered as one adapted to the general treatment of diseased antrum, but only in special cases when it is desirable to avoid the sacrifice of a tooth if possible.

### "A SIMPLIFIED FRACTURE SPLINT."

BY DR. JULES J. SARRAZIN.

He described a splint for a fractured inferior maxilla. Having secured a good model of the upper teeth, and also an accurate model of the lower teeth, break the lower cast at the place where the fracture exists, occlude the teeth and mount in an articulator. Make a simple rubber splint occluding perfectly with the upper teeth and extending lower on the gum than the exposed necks of the teeth, making the edges that rest on the gum well rounded and polished. Try in the mouth and make sure that with the jaws closed and pressed together all parts will go in place nicely and that the occlusion of the upper teeth and the splint will be perfect. Having ready sufficient bandage, that will not stretch, to wrap eight or ten times around the head and closed jaw, cheeks and ears of the patient, rapidly mix oxy-phosphate cement to a medium thick consistency, fill the inside of the splint with it, dry the lower teeth and jaw and insert the splint, bringing the splint and enclosed teeth to occlusion with the upper jaw. Bandage securely, fasten with safety pins and wait a little longer than necessary for the cement to thoroughly harden. The cement will have been forced between the proximal surfaces of the teeth fastening the dried splint securely. At the next sitting remove the bandages, trim off the excess of cement and the patient can masticate on the rubber occlusal surface; appearance will not be ungainly nor speech difficult. Before inserting the splint it should have been partially sawed, in different places, so that when ready to remove after the case has healed, the ends of the sawed lines can be easily continued with the engine bur, and the splint removed in sections. The remaining cement between the teeth that is found difficult to remove may be treated with strong alkaline baths until it dissolves.

DISCUSSION.

DR. P. J. FRIEDRICHS said that he had been quite impressed with Dr. Sarrazin's idea, but that he had not yet had a case where it would be applicable. He thought the idea of using oxy-phosphate cement *as a fixature* was original; the only difficulty he could see was in its removal, as it seemed to be necessary to remove it in pieces, while in cases of excessive inflammation it was sometimes desirable to remove the splint entire, and replace it. But the cement would certainly keep the splint in place, and the parts in perfect apposition, allowing nature to do her work.

DR. WALKER said he had been trying to induce Dr. Sarrazin to show a sample splint, but that he objected to doing so, because having carried it in his overcoat pocket he had sat down upon it and it had broken in three pieces at the lines sawed partially through to show the method of removal. Though calculated to withstand the strain of the masseter muscles, it would not stand being sat down upon!

In answer to a question, Dr. Sarrazin stated that the splint covered the occlusal surface of the lower teeth, and that the upper teeth occluded with the surface of the splint.

### "A CASE IN ORAL SURGERY."

BY DR. K. C. YOUNG, ANNISTON, ALABAMA.

The patient when first seen was found sitting upright in a common split-bottom chair in a room in the rear part of a drug store, a young man some twenty-three years of age, wrapped in a sheet saturated with blood, and supporting in his hand a dangling, unshapely mass of bone and teeth; the whole side of his face torn out. He was an operator in a cotton mill two miles out in the country, from whence he had been brought in an open buggy over a rough country road, nothing having been done for him except a hypodermic injection of morphia and strychnine. He had climbed up a ladder to adjust a belt, one end of the ladder resting on a revolving shaft, the other on a slippery floor. The ladder had evidently slipped and he had fallen upon the machine with his mouth open. The right cheek was cut open from the inner canthus to the commissure of the lip, and from a point about over the glenoid cavity joining a cut about on a line with the alæ nasi; a deep cut straight across the upper lip just under the nose, and six or eight cuts or punctures upon the chin. The nose had a multiple fracture and was driven over to the left; the whole of the upper right maxillary bone had been cut and torn completely from the face and was hanging upon the chin dangling by a small connection of gum tissue not wider than a lady's thumb nail, just over the lateral and cuspid. The bone had to be held to keep it from swinging about at each movement of the head. The cleft extended from the median line between the centrals along the line of articulation with the opposite maxillary, thence along the naso-maxillary articulation to floor of orbit, thence across to malo-maxillary articulation, thence to spheno-maxillary foramen across hard palate from incisor foramen to articulation of palate bone with pterygoid process of sphenoid. Raising flaps of the wound in the cheek the optic nerves could be seen and touched just as it enters into the schlerotic tunic; passing the finger down the pharynx the pterygoid process could be felt, rough and jagged where the articulation had been torn loose. The floor of the orbit was in plain view. The wound having been made as with a blunt instrument, was more of a laceration and wrenching apart, dragging the vessels out to their utmost so that when they broke they contracted as though tension had been made; the arteries had so contracted that they could not be taken up.

The mill superintendent begged me to save the bone, which contained all the teeth on the right side from the central incisor to the third molar, and including the tuberosity.

After many futile attempts I got the parts together, preserving the facial aspect, which was not unlike putting together a Chinese puzzle. To hold the bone up two ligatures were used, caught in the gum over the first molar, carried through the cheek and tied outside just under the eye. A wire suture was passed through the edges of the wound on hard palate, thence between the teeth to the opposite side. Three wires held the bone laterally, a wire was also passed from cuspid to cuspid, the bone being thus held up in three ways. The lower incisors were knocked out or broken off and the process driven back so that the teeth pointed down the throat. This was replaced and held by wire. The bone of the nose was fractured in several places and the nose much displaced. After the fractures were reduced rubber tubes the size of the little finger and ribbed were inserted into the nostrils, to hold the parts in shape and allow the patient to breathe through the nose. Forty odd stitches were taken in the soft tissue of the nose. In the corner of the lip a piece of tissue the size of the rubber crown on a lead pencil was hanging by a thread as it were, and almost black, but this was put back in place and held with delicate stitches and served to restore the shape of the mouth. The external wounds were dressed with aristol, and a linen cloth smeared with simple cerate laid on, and absorbent cotton placed over this. The whole was done up with a Gibson bandage and instructions given to syringe the mouth three times a day with a 10 percent solution of boric acid.

After the fifth day the patient began to improve rapidly and made a good recovery coming to the office after the third week for treatment. The external wounds left a wonderfully small amount of eschar, the nose was perfect and he uses his teeth as perfectly as though they had never been out of his head. The teeth are sound and seem to be alive, with the exception of the third molar which exfoliated. There is a small opening in the hard palate which will probably fill up in time. It has been greatly reduced by paring the edges of the break stimulating granulation.

This case shows the wonderful recuperative power of the facial region. The ingenuity of manipulation, the familiarity with the minute anatomy of the parts, the realization of the necessity of strict attention to aseptics, fits the dental surgeon very peculiarly to attend to this class of accidents.

DR. WALKER stated that he had seen the patient some months after the accident, and that although the face was somewhat scarred it was not badly; there was still a small opening in the hard palate which had not quite filled up.

Orthodontia and oral surgery passed.

